# The difficulties of conducting maternal death reviews in Malawi

**DOI:** 10.1186/1471-2393-8-42

**Published:** 2008-09-11

**Authors:** Eugene J Kongnyuy, Nynke van den Broek

**Affiliations:** 1 Child and Reproductive Health Group, Liverpool School of Tropical Medicine, Liverpool, UK

## Abstract

**Background:**

Maternal death reviews is a tool widely recommended to improve the quality of obstetric care and reduce maternal mortality. Our aim was to explore the challenges encountered in the process of facility-based maternal death review in Malawi, and to suggest sustainable and logically sound solutions to these challenges.

**Methods:**

SWOT (strengths, weaknesses, opportunities and threats) analysis of the process of maternal death review during a workshop in Malawi.

**Results:**

*Strengths*: Availability of data from case notes, support from hospital management, and having maternal death review forms. *Weaknesses*: fear of blame, lack of knowledge and skills to properly conduct death reviews, inadequate resources and missing documentation. *Opportunities*: technical assistance from expatriates, support from the Ministry of Health, national protocols and high maternal mortality which serves as motivation factor. *Threats*: Cultural practices, potential lawsuit, demotivation due to the high maternal mortality and poor planning at the district level. *Solutions*: proper documentation, conducting maternal death review in a blame-free manner, good leadership, motivation of staff, using guidelines, proper stock inventory and community involvement.

**Conclusion:**

Challenges encountered during facility-based maternal death review are provider-related, administrative, client related and community related. Countries with similar socioeconomic profiles to Malawi will have similar 'pull-and-push' factors on the process of facility-based maternal death reviews, and therefore we will expect these countries to have similar potential solutions.

## Background

The World Health Organisation (WHO) estimates that worldwide 536,000 women die each year from complications of pregnancy and childbirth [1]. More than 99% of these deaths occur in resource-poor countries [2]. Avoiding maternal deaths is possible even in resource-limited countries, but requires the right kind of information on which to base programmes. Knowing the level of maternal mortality is not enough to prevent further deaths; there is need to understand the underlying factors that led to the deaths. Each maternal death has a story to tell and can provide us with practical ways of addressing the problem. The term maternal death audit (MDA) is used to describe three approaches used to study the causes and characteristics of maternal deaths [3]. These approaches are confidential enquiry into maternal deaths (CEMD), facility-based death reviews and community-based death reviews (also called verbal autopsy) [4-8].

**Figure 1:**
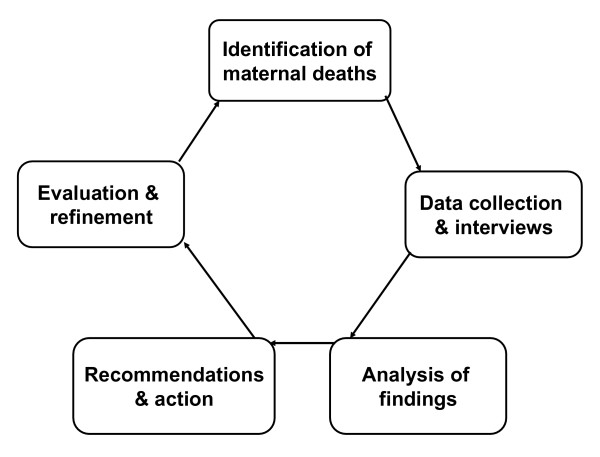
**Cycle of maternal death audit**. Maternal death audit (reviews) process consists of five steps. (a) Identification of maternal deaths: this can be difficult where many deaths take place outside health facilities. Even in health facilities, maternal deaths in other wards other than the maternity ward can be missed. (b) Data collection: data can be collected from many sources such as hospital registers, case notes, referral letters and interviews of family members and relatives. (c) Analysis of findings: data is analysed to identify the causes of maternal deaths and avoidable factors. (d) Recommendations and actions: recommendations are made to implement changes that will prevent the occurrence of similar deaths in the future. (e) Evaluation and refinement: the implementation of recommendations is followed up and evaluated and professional practice refined if necessary.

The World Health Organisation defines facility-based maternal death review as a "qualitative, in-depth investigation of the causes of, and circumstances surrounding, maternal deaths which occur in health care facilities." [3]. Gathering information from health professionals and relatives about the circumstances surrounding maternal deaths takes skills and sensitivity. Case notes and people's memories of events contain valuable information that can help improve the quality of care, and should be used appropriately. Maternal death review is based on the surveillance cycle which consists of identification of maternal deaths, data collection and interviews, analysis of findings, recommendations and action, evaluation and refinement (Figure 1).

There are many challenges involved in conducting facility-based maternal death reviews [3]. First of all, data may be missing due to poor documentation of case notes. This is particularly a big problem in developing countries where there are shortages of staff to record every event that takes place during delivery of care. The quality of case notes is an important determinant of the information required upon which to base the final recommendations. Secondly, data regarding community factors leading to the woman's death in the facility may be difficult to obtain. Thirdly, facility-based maternal death review can generate large volume of information that can be difficult to interpret and synthesize. Fourthly, facility-based maternal death reviews are sometimes not conducted in a blame-free manner. The findings from maternal death reviews are sometimes used by managers to punish those who provided the care. Under these circumstances, maternal death review is seen as means to obtain information to discipline the providers. Furthermore correct information may not be obtained especially when maternal death review is seen as a threat by those who took part in the management of the woman who died.

Malawi is one of the countries that have adopted the WHO recommendation of combining community-based and facility-based maternal death reviews to improve professional practice and reduce maternal mortality [8,9]. In addition, confidential enquiries are conducted regularly by the Malawi Ministry of Health. The Malawi Ministry of Health developed three forms which are currently used for maternal death review: (a) Maternal Death Notification Form contains the particulars of the deceased and its purpose is to notify the District Health Office within seven days of the maternal death, (b) Maternal Death Review Form is filled during maternal death review meetings and contains details of the causes of maternal death, factors that contributed to the maternal death, and recommendations made during maternal death review, and (c) Maternal Death Follow-up Form is used to follow-up the implementation of recommendations made during the maternal death reviews.

In Malawi maternal death reviews both facility- and community-based maternal death reviews are conducted at district level on monthly basis. One or two representatives are usually sent by each health facility to the District Health Office to attend the monthly maternal death reviews. Many cases of maternal death occur each month so that the District Maternal Death Review Committees are unable to review all these cases. The Ministry of Health therefore encourages every hospital to individually review all maternal deaths that occur in their facility. Despite the urge from the Ministry of Health, a few hospitals actually carry out maternal death reviews at facility level.

In 2005 the Ministry of Health developed forms for Maternal Death Review and introduced maternal death reviews in hospitals in Malawi. In 2006 an assessment of nine hospitals in three districts in Central Malawi revealed that only one hospital actually used the Maternal Death Review Follow-up Forms provided by the Malawi Ministry of Health [10]. In addition, many of the recommendations made during maternal death reviews were not implemented. In some cases maternal death reviews were not conducted in a blame-free manner and sub-optimal quality of care was prevalent among all the hospitals [11]. Following this assessment facility-based maternal death review was introduced in nine hospitals in 2006. It was clear the health care providers were facing many challenges at different levels of the maternal death review cycle.

The objective of this study was to explore the challenges encountered in the process of facility-based maternal death review in Malawi, and to suggest sustainable and logically sound solutions to these challenges.

## Methods

This study presents the results of SWOT (strengths, weaknesses, opportunities and threats) analysis conducted in February 2007 in Malawi (Lilongwe) to identify factors which facilitate or oppose maternal death review process. The workshop brought together experts in the fields of quality improvement, and maternal and neonatal health from within Malawi, and representatives of the quality improvement teams from nine hospitals in three districts (Lilongwe, Salima and Kasungu). These were all people directly involved in facility-based maternal death reviews. A total of 60 participants attended the workshop: 4 gynaecologist-obstetricians, 4 paediatricians, 2 public health experts, 2 general practitioners, 14 clinical officers and 34 midwives. In addition, there were three facilitators from the UK.

One hospital was found in Salima District (Salima District Hospital), one in Kasungu District (Kasungu District Hospital) and the rest in Lilongwe District. The 7 hospitals in Lilongwe included 1 central hospital, 2 Government community hospitals and 4 Mission hospitals.

Four key dimensions were studied: strengths and weaknesses were internal characteristics that facilitate and oppose the process of maternal death review, while opportunities and threats were external i.e. environmental factors that affect maternal death review process [8]. The facilitators divided a flipchart paper into fours squares and headed each square with one of the key headings. Ideas under each of the headings were discussed. Participants discussed how to exploit the strengths and opportunities to improve the maternal death review process, and how to overcome the weaknesses and threats in order to strengthen the maternal death review process.

The workshop was approved by the Reproductive Health Unit (RHU) of the Ministry of Health, Malawi.

## Results

### SWOT Analysis

The strengths, weaknesses, opportunities and threats encountered during the process of maternal death review are presented in Table 1.

**Table 1:** TOWS Matrix of the process of maternal death review in Malawi

	**Strengths** Having a task force (staff in maternity and female ward); Having standards to guide the Maternal Death Review Committee; Availability of data; Support from District Health Management Team; Availability of tools for maternal death review (review forms); Having financial resources for implementation; Having technical expertise; Knowing the evidence.	**Weaknesses** Fear of repercussions (blame); Other competing commitments; Some health care providers lack knowledge and skills; Inadequate resources (human and finances); Missing documentation (poor record keeping); Lack of transport to follow up at community level; Shortage of staff especially; senior staff such as Obstetricians & Gynaecologists; Shortages of drugs, supplies, blood etc.; Patients come from across the border or other districts.
**Opportunities** Technical assistance from international organisations; Support from the Ministry of Health; Having national safe motherhood protocols; Exchange visits to share ideas; Sharing experiences at workshops; Current high maternal mortality which provides the rationale to conduct maternal death reviews; Political will; Community support/involvement; Working with women's group.	**SO Strategy** Use standards and protocols to identify gaps in practice during maternal death reviews; Promote information documentation through the use of checklists and supportive supervision; Promote any forum of sharing experiences such as workshops and exchange visits; Involve senior management in maternal death reviews; Promote community involvement/support by working closely with women's group.	**WO Strategy** Use technical assistance from international organisations to improve knowledge and skills to conduct maternal death reviews; Promote forums for sharing experiences such as workshops and exchange visits; Use women's groups to advocate for more resources in maternal and neonatal health.

**Threats** Lack of openness on cultural practices; Threatening potential court case, Demotivation by the high maternal mortality; Political differences (parties); Communication problems and poor planning (district level reviews); Lack of political will; Shortages of supplies, drugs and blood; Shortages of human resources.	**ST Strategy** Promote community involvement by involving men and community leaders on maternity issues; Emphasize the principle of "no name, no blame" during maternal death reviews; Encourage the District Health Management Team to allocate resources for Maternal Death Reviews; Ensure proper stock inventory to prevent the frequent out-stocking of drugs, supplies and blood.	**WT Strategy** Promote community involvement by working with community leaders and women's group; Emphasize anonymity and confidentiality during maternal death reviews; Lobby for more staff from the Ministry of Health; Encourage districts to allocate resources for maternal and neonatal health when drawing their annual implementation plan.

**Strengths **were internal factors of each health facility that facilitated the process of maternal death review. The participants agreed that having qualified staff to review maternal deaths, case notes to provide information needed for the review and Maternal Death Review Forms to guide the review process have greatly facilitated maternal death review process at facility level. Support from the District Health Management Team (DHMT) is crucial for two reasons: it serves as a motivation for staff and facilitates the implementation of recommendations which need approval of resources from the management. Participants acknowledged the fact that support from DHTMs has been vital both in conducting maternal death reviews and in implementing recommendations from maternal death reviews. The nine hospitals have developed standards for maternal and neonatal care based on the national safe motherhood protocols. The health care providers refer to these standards whenever a disagreement rises during maternal death review; the standards have therefore been very helpful especially in facilities where maternal death review is conducted in the absence of senior staff such as doctors. Maternal death reviews are used to identify gaps in clinical practice, and recommendations are made after identification and discussion of avoidable factors that contributed to maternal death.

**Weaknesses **were internal barriers of each health facility that hinder the process of maternal death review. These weaknesses could be from the providers or from the management. They agreed that although there are attempts to ensure that maternal death review is conducted in a blame-free manner, there remains a bleak atmosphere of fear of repercussions (blame) among the providers. Moreover, due to shortage of staff, the providers conducting maternal death reviews have several other competing commitments which make it difficult to bring each and everyone involved in the management of the case together during the review. More so, conducting maternal death reviews at facility level is new to many providers who lack the knowledge and skills to properly review maternal deaths. Poor documentation which includes missing information from case notes and poor record keeping could flaw the conclusions from maternal death reviews and make any recommendations from such reviews to be irrelevant. Absence of senior staff such as doctors and specialists was identified as a major weakness during the process of maternal death review. Where reviews are conducted exclusively by junior staff, some provider-related factors are missed. Sometimes the recommendations made during maternal death reviews are not implemented due to inadequate resources (human and finances); this include lack of transport to follow up at community level and shortages of drugs, supplies, blood and human resources.

**Opportunities **were factors external to each facility that were likely to promote the process of maternal death review. The participants agreed that having national safe motherhood protocols was very useful; in fact the providers have made good use of the protocols by developing standards for maternal and neonatal care from these protocols and they refer to both standards and protocols whenever they are blocked during maternal death review. The providers have had several occasions when they met in a workshop to discuss and share their experiences of with staff from other hospitals. They found the sharing of experiences between health facilities a useful way of promoting and stimulating interest in maternal death reviews. Moreover, having external support from the Ministry of Health and from international organisations also boasted the morale of the providers and provided them with a pool of technical expertise to support them whenever they were in need. The current high level of maternal mortality was seen as a "push" factor that provides the rationale for maternal death reviews.

**Threats **were barriers external to each health facility that prevented the health care providers from successfully conducting maternal death reviews or implementing the recommendations from reviews. Cultural barriers could hinder the process of maternal death reviews or the implementation of recommendations from reviews. For example, in some areas women will not go to hospital when they are sick or in labour until they have permission from their husband who is not always there to give this permission. In addition, because of lack of openness on cultural practices, some factors might not be identified during maternal death review. For example, a woman in labour could be given traditional oxytocic drugs (herbal medicines to induce or augment labour) secretly by relatives without the knowledge of the health care providers. Traditional oxytocics frequently contribute to uterine rupture and maternal deaths. To convince women to stop using this oxytocic drug requires more than simple health education since it is rooted in the culture of the people. Law suits for poor management are now increasing in developing countries [11]. Health care providers might be afraid to reveal full information about the management of a woman who died during childbirth because of threatening potential court case. It was recognised that although the current high level of maternal mortality could be a motivation factor for maternal death reviews, it could also be a demotivation factor, especially is the providers do not find any progress despite their efforts to reduce maternal deaths.

### Strategies to address the difficulties of conducting maternal death reviews

The TOWS Matrix (Table 1) presents four conceptually distinct alternative strategies identified by the workshop participants. The participants recognized the fact that the four strategies overlap and recommended that they should be pursued concurrently as they strategies are not only complementary but also synergistic.

#### SO strategy

The aim of this strategy was to maximize both the strengths and opportunities. The participants identified the need to use standards and protocols to identify gaps in practice during maternal death reviews especially as it was not possible always to have senior technical staff during maternal death reviews. During the process of maternal death review, the junior staff should refer to standards on difficult issues where they fail to agree. Standards could guide them to reach a consensus and identify provider-related factors that contributed to maternal deaths. The participants also identified the need to promote information documentation and quality of data through the use of checklists and supportive supervision. Maternity staff could learn from each other by bringing them together to share their experiences during workshops and exchange visits. It was suggested public health nurses and Health Surveillance Assistants should work with women's groups to promote community involvement and support.

#### ST strategy

The strategy is based on the strengths of the health facilities that can deal with threats in the environment. The participants re-iterated the need to respect the principle of anonymity and confidentiality during maternal death reviews. All hospitals were encouraged to ensure proper stock inventory to prevent out-stocking of drugs, supplies and blood, while District Health Management Teams were encouraged to allocate resources for maternal death reviews when drawing their annual implementation plan. It was also noted that maternal death reviews was frequently forgotten when resources were being allocated. Health facilities order drugs and supplies regularly from the Central Drug Stores; they equally order blood from Malawi Blood Transfusion Services (MBTS) which collects blood centrally, screen, store and distribute to health facilities. Good prediction of weekly and monthly requirements combined with regular checking of what is in stock before placing a new order is important to prevent frequent under-supply or over-supply of drugs, supplies or blood. Involving men and community leaders in maternity issues was also emphasized.

#### WO strategy

This strategy attempts to minimize the weaknesses and to maximize the opportunities. Hospitals were encouraged to use the existing technical assistance from international organizations to build their capacity to conduct maternal death reviews. Any gaps identified during maternal death reviews should be filled by giving appropriate training to those concerned. Forums that promote sharing of knowledge and experiences such as workshops and exchange visits among staff of different health facilities could help build the capacity of maternity staff in conducting maternal death reviews. Women's group could be used to advocate for more resources for maternity care.

#### WT strategy

The aim of this strategy is to minimize the weaknesses and threats. Promoting community involvement, ensuring anonymity and confidentiality during maternal death reviews, encouraging districts to allocate resources for the implementation of recommendations from maternal death reviews and lobbying for more staff from the Ministry of Health were identified as strategies to address both the weaknesses and threats.

## Discussion

We have used SWOT analysis to explore the "push-and-pull" factors involved in the maternal death review process. To the best of our knowledge this is the first report on a structured analysis of the challenges encountered during maternal death review in a resource-limited country. The majority of studies have focused on how maternal death reviews are conducted and the number of cases reviewed [12-16]. This study approaches maternal death review from a different perspective – difficulties of conducting maternal death reviews and implementing recommendations from reviews.

The strength of this analysis lies on the fact that the health care providers identified the challenges as well as potential solutions themselves, as a way to promote ownership and sustainability. This can facilitate the implementation of decisions arrived at during the workshop. The use of a multidisciplinary team promoted team work and ensured that issues related to different disciplines were properly addressed.

Although most of these challenges were identified through SWOT analysis, it should be borne in mind that this list is not comprehensive and the list of potential solutions is not also comprehensive. For example, the health care provider can contribute in overcoming cultural barrier to maternal death review through advocacy. The provider through advocacy can change openness of cultural practices that prevents identification of community factors and political will. This was not mentioned by the participants during the workshop.

Good leadership and motivation of members of the Maternal Death Review Committee is essential to ensure continuity in maternal death reviews and to prevent demotivation that may result from high maternal mortality ratio. Although the health providers can have good ideas, these ideas will not be realized without support from the management. Some of the participants of this workshop were members of District Health Management Team such as the District Nursing Officers and District Health Officers.

Using SWOT analysis we identified many opportunities and challenges encountered during maternal death reviews in Malawi. The challenges are provider-related, administrative, client/family related and community related. In order to establish a successful maternal death review programme, these challenges should be taken into consideration as early as the conception phase. Potential solutions to challenges include proper documentation (e.g. using checklist and supportive supervision), emphasis of anonymity and confidentiality during maternal death review, building capacity of maternity staff to conduct maternal death reviews, good leadership, motivation of staff, using standards of care to guide the review committee, proper stock inventory, adequate resources, involvement of the community and support from the hierarchy. Countries with similar socioeconomic profiles to Malawi will have similar 'pull-and-push' factors on the process of facility-based maternal death reviews, and therefore we will expect these countries to have similar potential solutions.

## Competing interests

The authors declare that they have no competing interests.

## Authors' contributions

EJK: Conception, design, drafting of the protocol, analysis, interpretation and writing-up of all versions of the manuscript. NVDB: Reviewed the manuscript for important intellectual content. All authors read and approved the final manuscript. EKJ: is the guarantor.

## Pre-publication history

The pre-publication history for this paper can be accessed here:


